# Transformation leadership with knowledge sharing and employee career growth: the role of self-efficacy and psychological capital

**DOI:** 10.3389/fpsyg.2025.1624245

**Published:** 2025-10-31

**Authors:** Yun Bai

**Affiliations:** School of Education, The University of North Carolina at Chapel Hill, Chapel Hill, NC, United States

**Keywords:** transformation leadership, knowledge sharing, employee career growth, self-efficacy, psychological capital

## Abstract

Existing research lacks a systematic exploration of the relationships among transformational leadership, knowledge sharing, and employee career growth, as well as the mechanisms of self-efficacy and psychological capital in this context, especially the integrated analysis of mediating and moderating effects. This study aims to fill this gap by constructing a structural equation model based on social exchange theory and comprehensively applying qualitative and quantitative research methods. It deeply analyzes how transformational leadership promotes employee career growth through knowledge sharing and reveals the moderating and strengthening roles of self-efficacy and psychological capital. Based on 412 valid questionnaires and 15 in—depth interview data, methods such as descriptive statistics, regression analysis, mediating effect test, and moderating effect test were used to systematically verify the internal relationships among variables, providing new theoretical perspectives and empirical support for organizational management practices. The study found that transformational leadership has a significant positive impact on employee career growth (*β* = 0.603, *p* < 0.001), and knowledge sharing plays a partial mediating role between them (mediating effect value = 0.1505); self-efficacy significantly moderates the relationship between transformational leadership and knowledge sharing (*β* = 0.412, *p* < 0.001), and the mediating effect of knowledge sharing gradually weakens as the level of self-efficacy increases (low level: 0.0994; high level: 0.0615); psychological capital strengthens the positive relationship between transformational leadership and knowledge sharing (*β* = 0.422, *p* < 0.001) and enhances the mediating effect of knowledge sharing (low level: 0.1094; high level: 0.0715). Theoretically, this study enriches transformational leadership theory, deepens the understanding of the mediating mechanism of knowledge sharing, and expands the application boundaries of self-efficacy and psychological capital in organizational behavior. Practically, it suggests that enterprise managers should pay attention to cultivating transformational leadership styles, especially by improving leadership effectiveness through the four dimensions of moral example, visionary motivation, individualized consideration, and leadership charisma.

## 1 Introduction

In today’s rapidly changing business environment, employee career development has become the core driving force for organizational progress. Transformational leadership can stimulate employees’ internal motivation and promote their career growth, and knowledge sharing is also regarded as a key factor in promoting employee career development. Although existing academic research has confirmed that transformational leadership and knowledge sharing have a positive impact on employee career development, the research on the specific mechanism of action among transformational leadership, knowledge sharing, and employee career growth is still relatively scarce. At the same time, the influence of employees’ psychological resources such as self-efficacy and psychological capital in this process also needs to be further explored. This study aims to solve the following key problems: Does knowledge sharing play a mediating role between transformational leadership and employee career growth? What are the moderating and strengthening roles of self-efficacy and psychological capital in this relationship? The main deficiencies in existing research are that most previous studies only focused on the pairwise relationships among transformational leadership, knowledge sharing, and employee career growth, lacking a systematic exploration of the overall mechanism of action of the three. Moreover, there is little research on the influence of employees’ psychological resources in this process, and the complex influence of employees’ individual psychological factors on career growth has not been fully revealed. The value of this study lies in constructing a theoretical model covering transformational leadership, knowledge sharing, and employee career growth. By revealing the mediating role of knowledge sharing, it deeply analyzes the internal path through which transformational leadership affects employee career growth. At the same time, by studying the moderating and strengthening roles of self-efficacy and psychological capital, it further refines the mechanism of action of employees’ psychological resources in the relationship between leadership behavior and career growth. This not only enriches the theoretical research in the fields of leadership, knowledge management, and career development but also provides scientific basis and practical guidance for organizational managers to optimize leadership styles, improve knowledge management mechanisms, and thus better promote employee career development and organizational progress.

## 2 Research hypothesis and research model

### 2.1 Research hypothesis

#### 2.1.1 The relationship between change leadership and employee career growth

Transformational leadership is a leadership style centered on motivating, inspiring, and empowering employees, characterized by idealized influence, inspirational motivation, intellectual stimulation, and individualized consideration. This leadership style not only focuses on employees’ short-term performance but also emphasizes their long-term career growth, which refers to the continuous improvement in employees’ career capabilities, opportunities, and satisfaction (Ng and Feldman, 2014). Gardner and Avolio (1998) noted that transformational leadership fosters employees’ self-driven abilities by setting challenging goals and creating a positive atmosphere, thereby injecting sustained momentum into their career growth. Muhammad et al. (2025) further emphasized that leaders, through idealized influence and charismatic leadership, subtly influence employees, encouraging them to learn and emulate, thus enhancing their career capabilities. Rayees and Makhmoor (2025) argued that inspirational motivation helps clarify career direction, while individualized consideration addresses individual differences, both of which jointly promote career growth. Fawad et al. (2025) proposed that transformational leadership enhances employees’ confidence and sense of responsibility, enabling them to tackle more challenging tasks and adopt a more proactive attitude toward their career development. Based on this, the following hypotheses are proposed:

H1: Transformational leadership can significantly and positively drive employees’ career growth.

H1a: Leaders’ behaviors of idealized influence can significantly and positively promote employees’ career growth.

H1b: Leaders’ charismatic traits can significantly and positively facilitate employees’ career growth.

H1c: Leaders’ inspirational motivation measures can significantly and positively influence employees’ career growth.

H1d: Leaders’ individualized consideration behaviors can significantly and positively drive employees’ career growth.

#### 2.1.2 The relationship between transformational leadership and knowledge sharing

Knowledge sharing refers to the process by which individuals or teams actively transfer, exchange, and integrate explicit and implicit knowledge within an organization. Transformational leadership plays a critical role in promoting knowledge sharing by building trust, fostering collaboration, and providing support to motivate employees to share knowledge willingly. Social exchange theory provides theoretical support for this relationship, suggesting that transformational leadership enhances employees’ willingness to share knowledge by offering emotional support, resource guarantees, and role modeling (Blau, 1964). Garima et al. (2025) indicated that transformational leadership, through idealized influence, sets an example for employees, earning their respect and trust, and attracting their followership through charismatic leadership, thereby significantly enhancing their willingness to share knowledge. Danina et al. (2025) found that transformational leadership, through inspirational motivation, clarifies the organization’s future direction, inspiring employees to actively engage in knowledge exchange to achieve common goals. At the same time, individualized consideration makes employees feel valued, reducing their concerns about sharing and promoting knowledge-sharing behaviors. Ngoc and Ba (2025) further proposed that transformational leadership focuses on building emotional connections with employees, enhancing their trust and sense of belonging, making them more willing to share knowledge in a secure environment. Kumar and Rajeev (2025) emphasized that leaders, by leading by example and actively participating in sharing activities, provide behavioral models for employees, enabling them to improve the efficiency of knowledge dissemination and exchange through observational learning. Based on the above research, the following hypotheses are proposed:

H2: Transformational leadership can significantly and positively stimulate employees’ knowledge-sharing behaviors.

H2a: Leaders’ idealized influence behaviors can significantly and positively promote employees’ engagement in knowledge-sharing activities.

H2b: Leaders’ charismatic traits can significantly and positively encourage employees to participate in knowledge sharing.

H2c: Leaders’ inspirational motivation measures can significantly and positively motivate employees to engage in knowledge sharing.

H2d: Leaders’ individualized consideration behaviors can significantly and positively inspire employees to share knowledge.

#### 2.1.3 The relationship between knowledge sharing and employee career growth

Knowledge sharing is a critical factor influencing employee career development in modern organizations. By facilitating information exchange, skill enhancement, and collaborative innovation, it creates more career opportunities for employees (Cabrera and Cabrera, 2005). Social exchange theory posits that knowledge sharing, as a reciprocal behavior, strengthens trust and cooperation among employees, thereby supporting their career development (Blau, 1964). Fu et al. (2025) noted that knowledge sharing can significantly enhance employees’ career capabilities, enabling them to remain competitive in a rapidly changing workplace. Truong et al. (2025) found that knowledge sharing, by improving employees’ information acquisition abilities, can effectively drive their career growth. Menon (2025) further pointed out that knowledge sharing, by promoting collaboration and communication among employees, can significantly enhance their career development opportunities. Khan (2025) demonstrated that knowledge sharing, by boosting employees’ confidence and sense of responsibility, can effectively propel their career growth. From an information acquisition perspective, knowledge sharing creates a broad platform for information exchange, enabling employees to break through their personal knowledge limitations and access richer and more diverse industry trends, market developments, and technological innovations, thereby laying a solid foundation for their career development. The following hypotheses are proposed:

H3: Knowledge-sharing behaviors can positively promote employees’ career growth.

H3a: Employees’ knowledge collection behaviors can positively drive their career growth.

H3b: Employees’ knowledge contribution behaviors can positively promote their career growth.

#### 2.1.4 The mediating role of knowledge sharing

In the field of organizational management, knowledge sharing plays a crucial mediating role between transformation leadership and employee career development. Transformation leadership creates a positive organizational atmosphere with its unique style, effectively promoting knowledge sharing and providing opportunities for employees to learn and grow. In such a positive environment, employees actively share knowledge, gain exposure to diverse skills, enhance their professional abilities, and thus drive career advancement, such as promotions or salary increases. Sommerfeld and Park (2025) empirically demonstrated that the characteristic behaviors of transformation leadership stimulate employees ‘enthusiasm for knowledge sharing, which significantly mediates its impact on employee career growth. Huang et al. (2025) pointed out that under the encouragement of transformation leadership, knowledge sharing enhances employees’ information acquisition capabilities, improves work performance, and promotes career growth. Helalat et al. (2025) believe that the culture of knowledge sharing advocated by transformation leadership fosters employee collaboration and communication, develops teamwork and communication skills, and brings more career development opportunities. Chick (2025a) found that employee participation in knowledge sharing can boost confidence and responsibility, encouraging them to engage positively in their work and pursue career advancement. Based on this, the following hypothesis is proposed:

H4: Knowledge sharing plays a mediating role in the relationship between transformation leadership and employee career growth.

H4a: Knowledge collection plays a mediating role in the relationship between transformation leadership and employee career growth.

H4b: Knowledge contribution plays a mediating role in the relationship between transformation leadership and employee career growth.

#### 2.1.5 The moderating effect of self-efficacy

Self-efficacy, as an individual’s belief assessment of their ability to complete specific tasks, has an important impact on organizational behavior and plays a key moderating role in employees’ behavior. Employees with high self-efficacy are often more confident, actively take on challenges, and are actively involved in work; while those with low self-efficacy are prone to self-doubt and tend to retreat in the face of difficulties. In the context of knowledge sharing, self-efficacy significantly affects employees’ participation willingness and degree: employees with high self-efficacy believe that they can provide valuable knowledge and expect positive feedback, so they are more willing to participate in sharing; employees with low self-efficacy often avoid sharing due to doubts about the quality of their own knowledge. Transformational leadership can effectively improve employees’ self-efficacy by identifying employees’ strengths, providing positive feedback, and incentives. Dilliott et al. (2025) and Chick (2025b) found that self-efficacy plays a moderating role between transformational leadership and knowledge sharing. Employees with high self-efficacy are more actively involved in knowledge sharing under the influence of leadership, while employees with low self-efficacy need additional incentives. Peng et al. (2025) and Dilliott et al. (2025) pointed out that the enhancement of self-efficacy can improve employees’ confidence and sense of responsibility, promoting them to actively share knowledge. Jorge et al. (2025) and Peng et al. (2025) emphasized that employees with high self-efficacy are more inclined to cooperate and believe that their knowledge can create value for the team. Meier (2025) and Jorge et al. (2025) believed that it provides internal motivation for knowledge sharing by enhancing confidence and sense of responsibility, thereby promoting team innovation. Based on the above research, the following hypotheses are proposed:

H5: Self-efficacy will moderate the relationship between transformational leadership and knowledge sharing, that is, the level of self-efficacy will affect the degree of influence of transformational leadership on knowledge sharing.

H6: Self-efficacy will moderate the mediating effect of knowledge sharing in the relationship between transformational leadership and employee career growth. The strength of the mediating role of knowledge sharing will vary with different levels of self-efficacy.

H5: Self-efficacy plays a moderating role in the relationship between transformation leadership and knowledge sharing.

H6: Self-efficacy moderates the mediating effect of knowledge sharing.

#### 2.1.6 The strengthening effect of psychological capital on the relationship between transformation leadership, knowledge sharing and employee career growth

Psychological capital is a crucial psychological resource that influences employee behavior. Research has shown that psychological capital can strengthen the relationship between transformation leadership and knowledge sharing, thereby promoting employees ‘career development. For example, Mao et al. (2025) and Meier (2025) pointed out that psychological capital plays a significant reinforcing role in the relationship between transformation leadership and knowledge sharing. An et al. (2025) and Mao et al. (2025) also found that psychological capital enhances employees’ confidence and sense of responsibility, effectively promoting their knowledge-sharing behavior. Hoang et al. (2025) and An et al. (2025) further noted that psychological capital promotes cooperation and communication among employees, significantly enhancing their knowledge-sharing capabilities. Adotey et al. (2025) and Hoang et al. (2025) research indicates that psychological capital, by boosting employees’ confidence and sense of responsibility, can effectively promote their knowledge-sharing behavior. Based on this, the following hypothesis is proposed:

H7: Psychological capital plays a reinforcing role in the relationship between transformation leadership and knowledge sharing.

H8: Psychological capital plays a reinforcing role in the mediating effect of knowledge sharing (Table 1).

**Table 1:** Summary of research hypotheses.

Number	Hypothesis content
H1	Transformational leadership can significantly and positively promote the process of employees’ career growth.
H1a	Leaders’ behavior of setting an example through virtue can significantly and positively promote employees’ career growth.
H1b	The leadership charm traits possessed by leaders can significantly and positively contribute to employees’ career growth.
H1c	The vision—inspiring measures implemented by leaders can significantly and positively influence employees’ career growth.
H1d	The personalized care behavior given by leaders to employees can significantly and positively promote employees’ career growth.
H2	Transformational leadership can significantly and positively stimulate employees’ knowledge—sharing behavior.
H2a	Leaders’ behavior of setting an example through virtue can significantly and positively promote employees’ knowledge—sharing activities.
H2b	The leadership charm traits possessed by leaders can significantly and positively drive employees to participate in knowledge sharing.
H2c	The vision—inspiring measures implemented by leaders can significantly and positively encourage employees to engage in knowledge sharing.
H2d	The personalized care behavior given by leaders to employees can significantly and positively motivate employees to engage in knowledge sharing.
H3	Knowledge—sharing behavior can positively promote employees’ career growth.
H3a	Employees’ behavior of knowledge collection can positively promote their own career growth.
H3b	Employees’ behavior of knowledge contribution can positively promote their own career growth.
H4	Knowledge sharing plays an intermediary role in the relationship between transformational leadership and employees’ career growth.
H4a	Employees’ knowledge collection behavior plays an intermediary role in the relationship between transformational leadership and employees’ career growth.
H4b	Employees’ knowledge contribution behavior plays an intermediary role in the relationship between transformational leadership and employees’ career growth.
H5	Self-efficacy will moderate the relationship between transformational leadership and knowledge sharing, that is, the level of self-efficacy will affect the degree of the impact of transformational leadership on knowledge sharing.
H6	Self-efficacy will moderate the mediating effect of knowledge sharing in the relationship between transformational leadership and employees’ career growth. The intensity of the mediating role of knowledge sharing will vary with different levels of self-efficacy.
H7	Employees’ psychological capital can strengthen the positive relationship between transformational leadership and knowledge sharing.
H8	Employees’ psychological capital can strengthen the mediating effect of knowledge sharing in the relationship between transformational leadership and employees” career growth.

### 2.2 Research model

**Figure 1:**
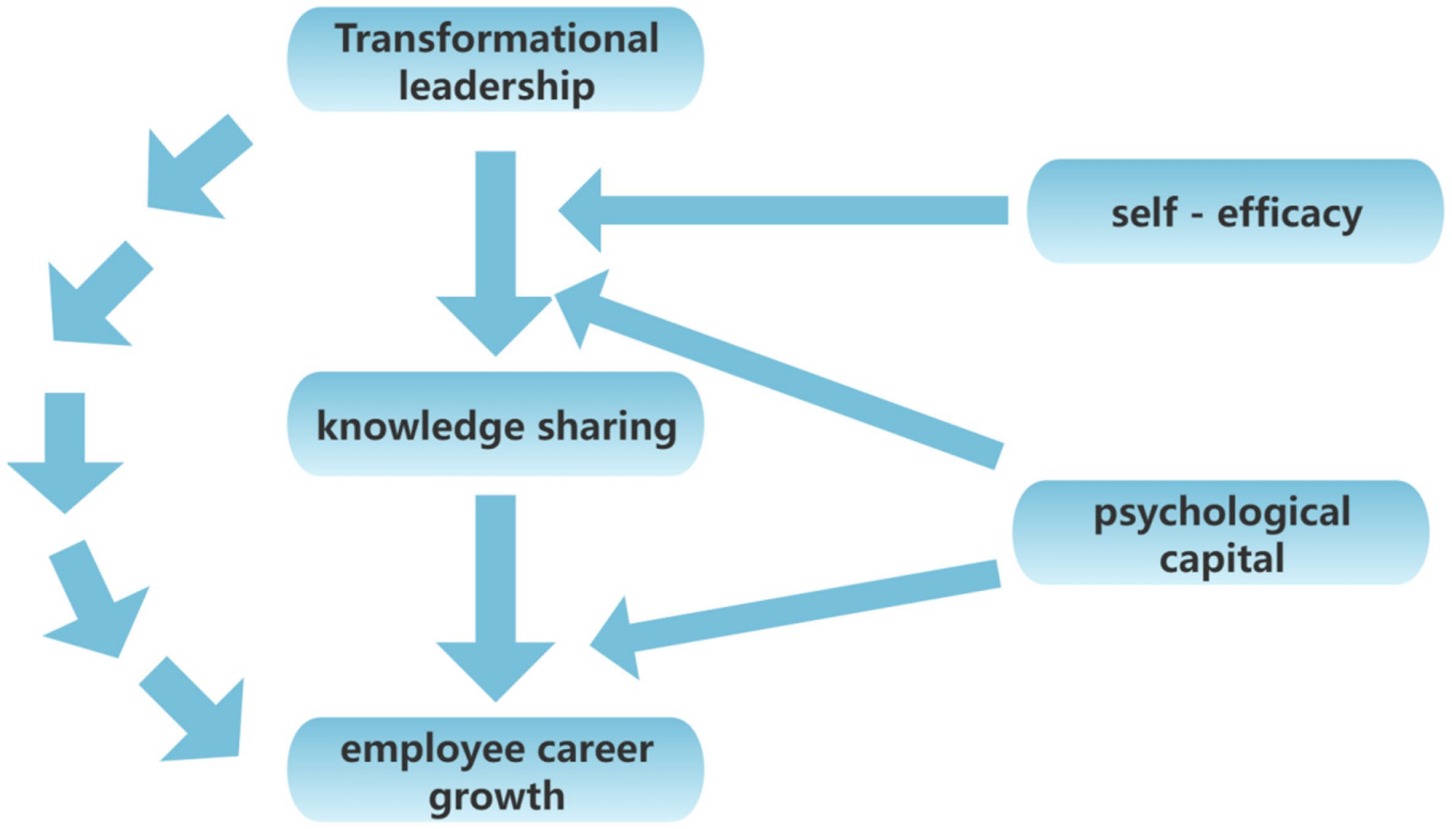
Hypothetical model of this study.

Based on the above assumptions, this study constructs a theoretical model linking transformation leadership, knowledge sharing, employee career development, self-efficacy, and psychological capital. As shown in Figure 1, transformation leadership influences knowledge-sharing behavior, thereby promoting employee career development. At the same time, self-efficacy and psychological capital play a moderating and reinforcing role in this process.

### 2.3 Data collection

#### 2.3.1 Data collection methods

This study uses the questionnaire survey method as the main data collection approach to obtain data on variables related to transformational leadership, knowledge sharing, employee professional development, self-efficacy, and psychological capital. The questionnaire design is based on mature scales at home and abroad and is appropriately adjusted according to the research background to ensure the validity and applicability of the content. The questionnaire is divided into five parts: The first part is the transformational leadership scale, covering four dimensions of moral example, leadership charisma, visionary motivation, and individualized consideration; the second part is the knowledge-sharing scale, consisting of two dimensions of knowledge collection and knowledge contribution; the third part is the employee career growth scale, which is used to evaluate employees’ growth and development in multiple aspects such as professional skills, career opportunities, and career achievements; the fourth part is the self-efficacy scale to evaluate employees’ confidence in their own abilities; the fifth part is the psychological capital scale, covering four dimensions of hope, resilience, optimism, and self-efficacy. The questionnaire uses a Likert 5-point scale (1 = strongly disagree, 5 = strongly agree) for measurement. To ensure the diversity and representativeness of the sample, the data collection not only covers many provinces in the country but also includes samples from other regions or countries, such as employees of enterprises in Southeast Asia, Europe, and North America, to test the cross—cultural applicability and generalizability of the research model. In addition, the study specifically focuses on the differences among different industries. For example, a comparative analysis is conducted between the technology industry (such as information technology and software development) and traditional manufacturing to explore the mechanism of action of variables such as transformational leadership, knowledge sharing, self-efficacy, and psychological capital in different industry backgrounds and their impact on employee career growth. Through this cross—regional and cross—industry comparative analysis, this study can not only verify the applicability of the theoretical model in different cultural and work environments but also provide more targeted guidance for management practices in different industries. The questionnaire is distributed through a combination of online platforms (such as Wenjuanxing and Tencent Questionnaire) and offline paper questionnaires, and a total of 500 valid questionnaires are recovered, and the sample size meets the requirements for structural equation model analysis.

### 2.4 Data analysis method

This study employs quantitative analysis methods for data analysis, which are specifically divided into the following steps. First, the SPSS 26.0 software package is utilized to conduct descriptive statistical analysis, describing basic information such as sample characteristics, variable means, and standard deviations. Then, the Cronbach’s *α* coefficient and exploratory factor analysis (EFA) are used to verify the reliability and validity of the scale, ensuring the scientificity and reliability of the measurement tool. Meanwhile, the AMOS 24.0 software is used to perform confirmatory factor analysis (CFA) to evaluate the model’s fitting effect and the discriminant ability between different variables. Subsequently, multivariate regression analysis is carried out to examine the direct effect of transformational leadership on knowledge sharing and employee career growth, as well as the mediating role of knowledge sharing. To diagnose the multicollinearity problem, a variance inflation factor (VIF) test is conducted, and all VIF values are below the threshold of 10, indicating no significant multicollinearity problem. Additionally, stratified regression analysis is adopted to explore the mediating roles of self-efficacy and psychological capital, revealing the complex interrelationships between variables. To ensure measurement transparency, the scale format and point settings are clearly described. To test the convergent validity, the average variance extracted (AVE) and composite reliability (CR) are calculated. All AVE values are greater than 0.5, and CR values are higher than 0.7, indicating good convergent validity of the scale. The Fornell—Larcker criterion is used to test the discriminant validity, ensuring that the square root of the AVE of each construct is greater than its correlation with other constructs.

## 3 Empirical analysis

### 3.1 Construction of structural equation model

This paper uses structural equation modeling (SEM) in the empirical part to test theoretical models and hypotheses. The variables involved in the model include transformation leadership (C), knowledge sharing (K), employee career growth (E), self-efficacy (S), and psychological capital (P). The following is the formula representation of the model:

(1) Direct effect model. The employee career growth (E) is directly affected by the transformation leadership (C), which can be expressed by the following linear regression equation:

(1) E=α+βC+ε

among,

α is the intercept term.

β is the regression coefficient.

ε is the error term.

(2) Mediation effect model. Knowledge sharing ( K ) plays a mediating role between transformation leadership ( C ) and employee career growth ( E ). The model can be divided into two equations:

(2) $$K=\alpha_{1}+\beta_{1}C+\varepsilon_{1}$$

(3) $$E=\alpha_{2}+\beta_{2}C+\mathrm{\gamma K}+\varepsilon_{2}$$

Among them, $\beta_{1}$ represents the influence of transformation leadership on knowledge sharing, γ represents the influence of knowledge sharing on employees’ career growth, and $\beta_{2}$ represents the direct influence of transformation leadership on employees’ career growth.

(3) Moderation effect model. Self-efficacy ( S ) moderates the relationship between transformation leadership ( C ) and knowledge sharing ( K ), which can be expressed by the following equation:

(4) $$K=\alpha_{3}+\beta_{3}C+\gamma_{3}S+\delta (C\times S)+\varepsilon_{3}$$

Among them δ , the coefficient indicating the moderating effect, that is, the interaction term between self-efficacy and transformation leadership on knowledge sharing.

(4) Reinforcement role model. Psychological capital ( P ) plays a reinforcing role in the relationship between transformation leadership ( C ) and knowledge sharing ( K ), which can be expressed by the following equation:

(5) $$K=\alpha_{4}+\beta_{4}C+\gamma_{4}P+\delta (C\times P)+\varepsilon_{4}$$

Among them δ , the coefficient representing the reinforcement effect, that is, the interaction term between psychological capital and transformation leadership on knowledge sharing.

(5) Comprehensive model. All variables and effects are considered comprehensively, and the model can be expressed as:

(6) $$K=\alpha_{5}+\beta_{5}C+\gamma_{5}S+\varepsilon_{5}E+\beta_{6}C+\gamma_{6}K+\delta_{6}S+\tau_{6}P+\varepsilon_{6}$$

Among them, each coefficient represents the direct and indirect influence of different variables on knowledge sharing and employee career growth. Through the above formula, the complex relationship between transformation leadership, knowledge sharing, self-efficacy, psychological capital and employee career growth can be systematically analyzed, and the proposed hypothesis can be verified.

### 3.2 Sample description

#### 3.2.1 Descriptive statistics of samples

A total of 500 questionnaires were distributed in this study, including 120 paper questionnaires and 380 online questionnaires. After checking, 88 invalid questionnaires were eliminated, and finally 412 valid questionnaires were recovered, with a recovery rate of 82.4% (Table 2).

**Table 2:** Basic characteristics of valid samples.

Category	Option content	Frequency	Percentage
Gender	Male	152	36.9%
	Female	260	63.1%
Age	25 years old and below	108	26.2%
	26–35 years old	132	32.0%
	36–45 years old	126	30.6%
	46–55 years old	38	9.2%
	56 years old and above	8	1.9%
Education	Below junior college	42	10.2%
	Junior college	68	16.5%
	Undergraduate	220	53.4%
	Master	64	15.5%
	Doctorate and above	18	4.4%
Marital status	Unmarried	185	44.9%
	Married	227	55.1%
Years of service	Less than 1 year	112	27.2%
	1–3 years (including 1 year)	76	18.4%
	3–5 years (including 3 years)	58	14.1%
	5–10 years (including 5 years)	70	17.0%
	10–15 years (including 10 years)	54	13.1%
	15 years and above	42	10.2%
Management level	General employees	162	39.3%
	Grass—roots managers	98	23.8%
	Middle—level managers	92	22.3%
	Senior managers	60	14.6%
Monthly income level	Below 6,000 yuan	110	26.7%
	6,000–15,000 yuan (including 6,000)	156	37.9%
	15,000–30,000 yuan (including 15,000)	68	16.5%
	30,000–50,000 yuan (including 30,000)	36	8.7%
	Above 50,000 yuan	42	10.2%
Team size	5 people and below	82	19.9%
	6–10 people	148	35.9%
	11–15 people	48	11.7%
	16–20 people	44	10.7%
	21 people and above	90	21.8%
Organizational culture	Innovative	150	36.4%
	Traditional	120	29.1%
	Hybrid	142	34.5%
Company policy support	High support	180	43.7%
	Medium support	160	38.8%
	Low support	72	17.5%

In terms of gender distribution, females accounted for a larger proportion, at 63.1%, while males accounted for 36.9%. Regarding the age structure, the 26–45 age group was the main body, with those aged 26–35 accounting for 32.0% and those aged 36–45 accounting for 30.6%, indicating that middle—aged and young people were the main components of the sample. In the education distribution, undergraduates accounted for the largest proportion, reaching 53.4%.

In terms of marital status, married people accounted for 55.1%, slightly more than the unmarried. Regarding years of service, employees with less than 1 year of service had the highest proportion, at 27.2%. In terms of management level, general employees accounted for 39.3%, with a relatively large number, and the proportions of grass—roots, middle—level, and senior managers decreased in turn.

In the monthly income level, the 6,000–15,000 yuan range accounted for 37.9%, which was the range with the largest proportion, reflecting the concentrated trend of the sample income distribution. Regarding team size, teams with 6–10 people accounted for 35.9%.

In the organizational culture, innovative culture accounted for 36.4%, traditional culture accounted for 29.1%, and hybrid culture accounted for 34.5%. In terms of company policy support, high—support accounted for 43.7%, medium—support accounted for 38.8%, and low—support accounted for 17.5%.

#### 3.2.2 Variable descriptive statistics

This part of the study focuses on descriptive statistical analysis of variables such as transformation leadership, knowledge sharing, self-efficacy, psychological capital and employee career growth. The maximum value, minimum value, average value and standard deviation of each variable are calculated to comprehensively present the sample information (Table 3).

**Table 3:** Descriptive statistical analysis of each variable.

Variable	Number of cases	Minimum	Maximum	Mean	Standard deviation
Transformational leadership	412	1.05	5.23	3.9023	0.62103
Moral modeling	412	1.10	5.17	3.8872	0.73012
Visionary motivation	412	1.15	5.42	4.0521	0.64021
Individualized consideration	412	1.12	5.68	3.8215	0.76213
Leadership charisma	412	1.02	5.79	4.0620	0.63120
Employee career growth	412	1.55	5.36	3.7612	0.60123
Career goal progress	412	1.70	5.14	3.8642	0.67210
Career ability development	412	2.20	5.29	4.0715	0.65021
Promotion speed	412	1.00	5.58	3.5721	0.77123
Reward growth	412	1.00	5.47	3.4512	0.87210
Self-efficacy	412	2.05	5.62	3.8210	0.57123
Task Confidence	412	2.10	5.53	3.8521	0.58210
Goal—achievement confidence	412	2.00	5.24	3.8123	0.59210
Challenge—coping confidence	412	2.05	5.38	3.8012	0.60210
Knowledge sharing	412	2.45	5.71	4.1215	0.46210
Knowledge collection	412	2.60	5.49	4.1723	0.53120
Knowledge contribution	412	2.30	5.67	4.0612	0.53210
Knowledge application	412	2.50	5.26	4.1021	0.54210
Knowledge dissemination	412	2.40	5.59	4.0823	0.55210
Psychological capital	412	2.15	5.72	3.9123	0.58210
Self-confidence	412	2.20	5.63	3.9321	0.59210
Valid cases (column—wise)	412	–	–	–	–

This study conducted a descriptive statistical analysis of variables such as transformational leadership, knowledge sharing, self-efficacy, psychological capital, and employee career growth, and comprehensively presented the sample information by calculating the maximum, minimum, mean, and standard deviation of each variable. From the data, the sample size for each variable was 412 cases. From the perspective of transformational leadership, the overall mean was 3.9023, and the standard deviation was 0.62103. The means of visionary motivation and leadership charisma in each dimension were higher than 4.0521 and 4.0620, respectively. There were obvious differences in the standard deviations of moral modeling and individualized consideration, which were 0.73012 and 0.76213 respectively, indicating significant differences among different samples in these two aspects. The mean of employee career growth was 3.7612, and the standard deviation was 0.60123. The mean of career ability development was the largest, at 4.0715. The standard deviations of promotion speed and reward growth were relatively large, at 0.77123 and 0.87210 respectively, reflecting that employees’ growth differences in these two dimensions were affected by various factors. The mean of self-efficacy was 3.8210, and the standard deviation was 0.57123, indicating that the data distribution was relatively concentrated. The means of task confidence, goal—achievement confidence, and challenge—coping confidence were 3.8521, 3.8123, and 3.8012 respectively, indicating a relatively high level of self-efficacy in the sample. The mean of knowledge sharing was 4.1215, at a relatively high level. The means of knowledge collection and knowledge contribution in its dimensions were 4.1723 and 4.0612 respectively, indicating good knowledge—sharing performance in the sample. The mean of psychological capital was 3.9123, and the standard deviation was 0.58210, indicating a relatively concentrated data distribution and a relatively stable level of psychological capital among employees. Among them, the mean of self-confidence was 3.9321, indicating a relatively high level of psychological capital in the sample.

### 3.3 Reliability analysis

**Figure 2:**
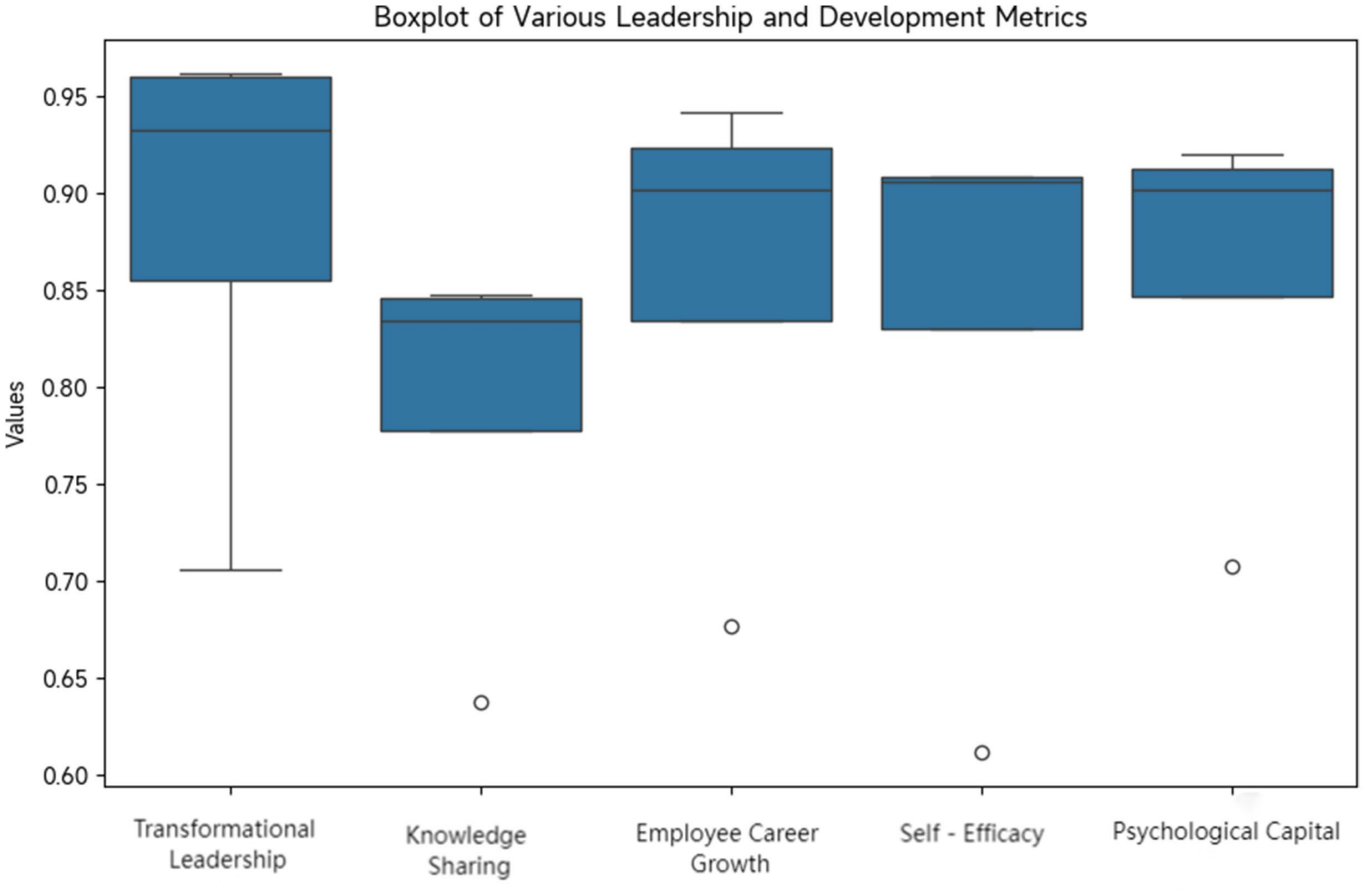
Distribution of reliability of each scale.

Reliability analysis mainly evaluates the reliability of the scale through the correlation statistical indicators of each variable items, such as CITC (correlation coefficient between items and total score), mean value of Cronbach’s *α* after item deletion, mean value of Cronbach’s α in each dimension and total Cronbach’s α (Table 4, Figure 2).

**Table 4:** Reliability analysis of variables.

Item	CITC	The average value of the deleted item, Cronbach’s α	Average of Cronbach’s α across dimensions	Total Cronbach’s α
Transformation leadership	0.706	0.960	0.905	0.962
Knowledge sharing	0.638	0.824	0.845	0.848
Employee career growth	0.677	0.917	0.887	0.942
Efficacy	0.612	0.903	0.909	0.909
Psychological capital	0.708	0.911	0.893	0.920

According to the data in Table 3, all primary indicators CITC are above 0.5, indicating good consistency between each item and the total score. This means that each item can reflect the overall characteristics of the variables. The average value of Cronbach’s *α* for all removed primary indicators is close to or slightly lower than the total Cronbach’s α, suggesting a balanced contribution of each topic to the reliability of the scale. Removing any single topic would not significantly increase the reliability of the scale.

All primary indicators have a mean Cronbach’s α above 0.7 across all dimensions, indicating good internal consistency within each dimension, meaning items are highly correlated within the same dimension. The total Cronbach’s α for all primary indicators is above 0.8, further confirming that the reliability of each scale is high and suitable for subsequent in-depth analysis (Figure 2).

### 3.4 Validity analysis exploratory factor analysis

Exploratory factor analysis primarily relies on the KMO (Kaiser-Meyer-Olkin) test, supplemented by Bartlett’s test, to evaluate the suitability of data for factor analysis. The KMO values for transformation leadership, knowledge sharing, employee career development, self-efficacy, and psychological capital are 0.951, 0.814, 0.903, 0.910, and 0.925, respectively. Generally, a KMO value closer to 1 is better; all these variables have KMO values above 0.8, indicating that the data is well-suited for factor analysis.

**Figure 3:**
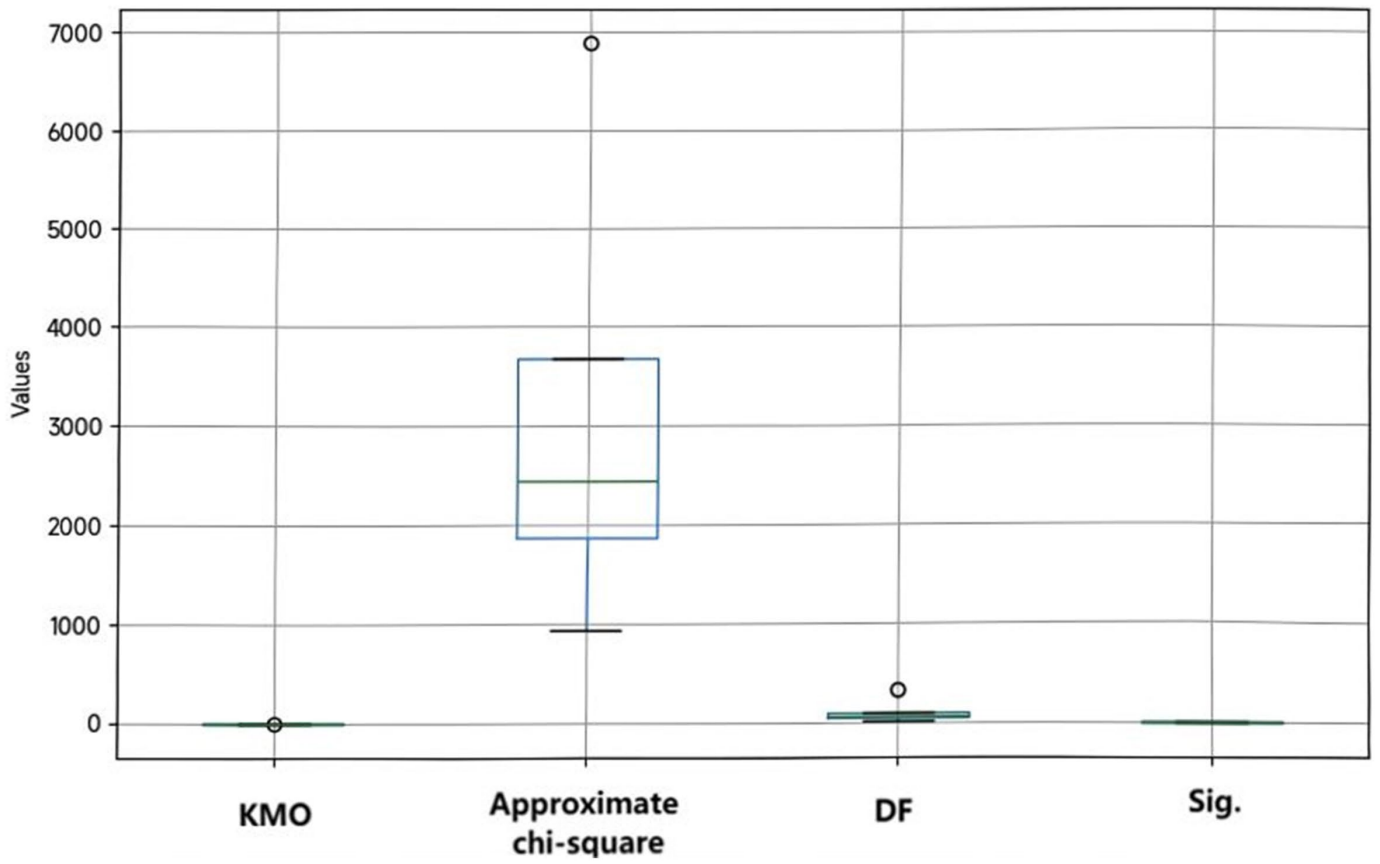
Exploratory factor validity analysis.

The approximate chi-square value of Bartlett’s test is large, and the Sig. value is 0.000, indicating that the correlation matrix is not a unit matrix. That is to say, there is significant correlation between variables. This further proves that the factor analysis is reasonable (Table 5, Figure 3).

**Table 5:** KMO and Bartlett’s test of exploratory factors.

Factor	KMO	Bartlett test		
		Approximate chi-square	df	Sig.
Transformation leadership	0.951	6886.030	325	0.000
Knowledge sharing	0.814	934.394	15	0.000
Employee career growth	0.903	3677.790	105	0.000
Efficacy	0.910	1864.375	45	0.000
Psychological capital	0.925	2450.620	66	0.000

### 3.5 Validity analysis confirmatory factor analysis

This section employs mature scales and uses the AMOS26.0 software package to conduct confirmatory factor analysis on indicators such as transformation leadership, self-efficacy, knowledge sharing, employee career development, and psychological capital. It also tests both absolute fit indices and relative fit indices to evaluate the model’s fit. The evaluation criteria for the model’s fit are set as follows: the closer CMIN/DF approaches 1, the better the model performance; when this value is between 1 and 3, it indicates a good fit; when between 3 and 5, it suggests an acceptable fit. RMR/RMSEA <0.05 means the model fits extremely well, while values between 0.05 and 0.08 are also acceptable. Indicators such as GFI, AGFI, NFI, and CFI greater than 0.9 indicate a good fit; values between 0.8 and 0.9 are still acceptable (Table 6).

**Table 6:** Evaluation of structural equation model fit.

Class	Evaluating indicator	Evaluation criterion
Absolute fit	CMIN/DF	1–3 fit well, the closer to 1 the better; 3–5 is acceptable
	RMR/RMSEA	<0.05 close to fit; 0.05–0.08 acceptable
Relative fitting	CFI/TLI/IFI/NFI	>0.9 is well fitted, the closer to 1 the better; 0.8–0.9 is acceptable

From the data in Table 6, it is evident that the five-factor model (transformation Leadership, Knowledge Sharing, Self-Efficacy, Employee Career Development, Psychological Capital) has the best fit. The Δχ2/df ratio is 3.521, which falls within the range of 3–5, indicating a reasonable fit; IFI = 0.945, TLI = 0.928, CFI = 0.943, all greater than 0.900, suggesting good fit; RMSEA = 0.049 < 0.08, indicating that the model closely matches the fit results.

**Figure 4:**
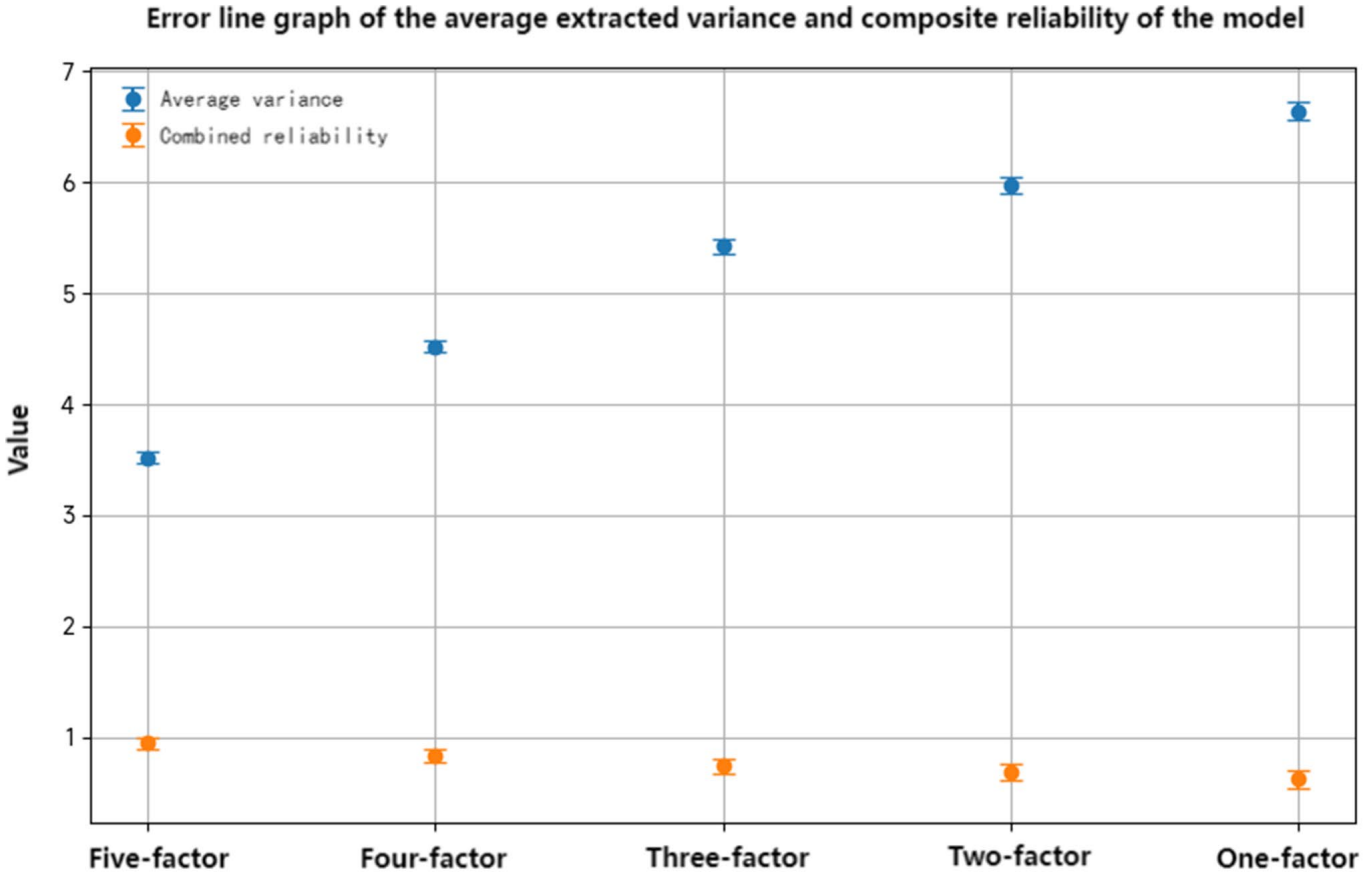
Average extraction variance and combined reliability error line of the model.

Compared to the five-factor model, the four-factor, three-factor, two-factor, and single-factor models all showed a significant decline in fit performance and successfully passed the chi-square test with a significance level of 0.001. This finding reveals a clear difference between other factor models and the five-factor model, which performs better in data interpretation and demonstrates high differentiation among variables. The average variance extracted by the models and the error line for combined reliability are shown in Table 7 and Figure 4.

**Table 7:** Comparison and analysis of overall measurement model and differentiation validity results.

Number	Model	χ2	df	χ2/df	IFI	TLI	CFI	RMSEA	Model comparison	Δχ2	Δdf
1	Five factors	5623.138	1,597	3.521	0.945	0.928	0.943	0.049	–	–	–
2	Four factors	7396.394	1,636	4.521	0.835	0.819	0.834	0.054	2 VS 1	1773.256 ***	39
3	Three factors	8875.124	1,638	5.421	0.742	0.723	0.740	0.065	3 VS 1	3251.986 ***	41
4	Two factors	9786.637	1,639	5.971	0.685	0.663	0.683	0.073	4 VS 1	4163.499 ***	42
5	Single factor	10896.739	1,640	6.643	0.621	0.598	0.619	0.081	5 VS 1	5273.601 ***	43

*** represents *p* < 0.001. (1) Five factors: transformation leadership, knowledge sharing, self-efficacy, employee career growth, and psychological capital. (2) Four factors: transformation leadership, knowledge sharing + self-efficacy, employee career growth, psychological capital. (3) Three factors: transformation leadership + knowledge sharing + self-efficacy, employee career growth, psychological capital. (4) Two factors: transformation leadership + knowledge sharing + self-efficacy + employee career growth, psychological capital. (5) Single factor: transformation leadership + knowledge sharing + self-efficacy + employee career growth + psychological capi.

### 3.6 Qualitative interview analysis

To further explore the in—depth relationships among the research variables, this study conducted semi—structured interviews with 10 employees and 5 managers, focusing on the moderating role of self-efficacy between transformational leadership, knowledge sharing, and employee career growth. The interview content revolved around “how self-efficacy affects knowledge—sharing behavior” and “how transformational leadership promotes employee career growth through self-efficacy.” The interview results are presented in Table 8.

**Table 8:** Qualitative analysis of interview results.

Theme	Representative views	Frequency	Typical quotations
Self-efficacy and knowledge sharing	Employees with high self—efficacy are more willing to share knowledge because they believe they have the ability to contribute to the team.	8/15	“I think I have the ability to help my colleagues solve problems, so I’m very willing to share my experience and knowledge.” (Employee A)
	Employees with low self-efficacy often fear sharing incorrect knowledge and worry about being questioned or criticized.	5/15	“I’m worried that the content I share is not accurate enough and may be questioned by my colleagues, so I rarely take the initiative to share.” (Employee B)
Transformational leadership and self-efficacy	Transformational leadership enhances employees’ self-efficacy through visionary motivation and individualized consideration.	10/15	“The leader often encourages me and says I’m very capable, which makes me more confident to try new tasks.” (Employee C)
	Leadership charisma and moral modeling make employees trust the leader more, thereby enhancing self-efficacy.	7/15	“The leader leads by example, which makes me feel that as long as I work hard, I can be as successful as him.” (Employee D)
Moderating role of self-efficacy	Self-efficacy plays a bridging role between transformational leadership and knowledge sharing. The leader’s support enhances employees’ willingness to share knowledge.	9/15	“The leader’s support makes me more confident, so I’m more willing to share my ideas in the team.” (Employee E)
	Employees with high self-efficacy are more likely to transform knowledge sharing into career—growth opportunities under transformational leadership.	6/15	“Because I believe in my ability, I regard knowledge sharing as an opportunity to improve my career development.” (Employee F)
Knowledge sharing and career growth	Knowledge sharing helps employees obtain more resources and information, thereby accelerating career growth.	12/15	“Through knowledge sharing, I’ve learned many new skills, which have made me perform better at work.” (Employee G)
	Knowledge sharing makes employees more influential in the team, thereby obtaining more promotion opportunities.	7/15	“Because I often share knowledge in the team, the leader thinks I have potential, so he recommended me for promotion.” (Employee H)

### 3.7 Correlation analysis

**Figure 5:**
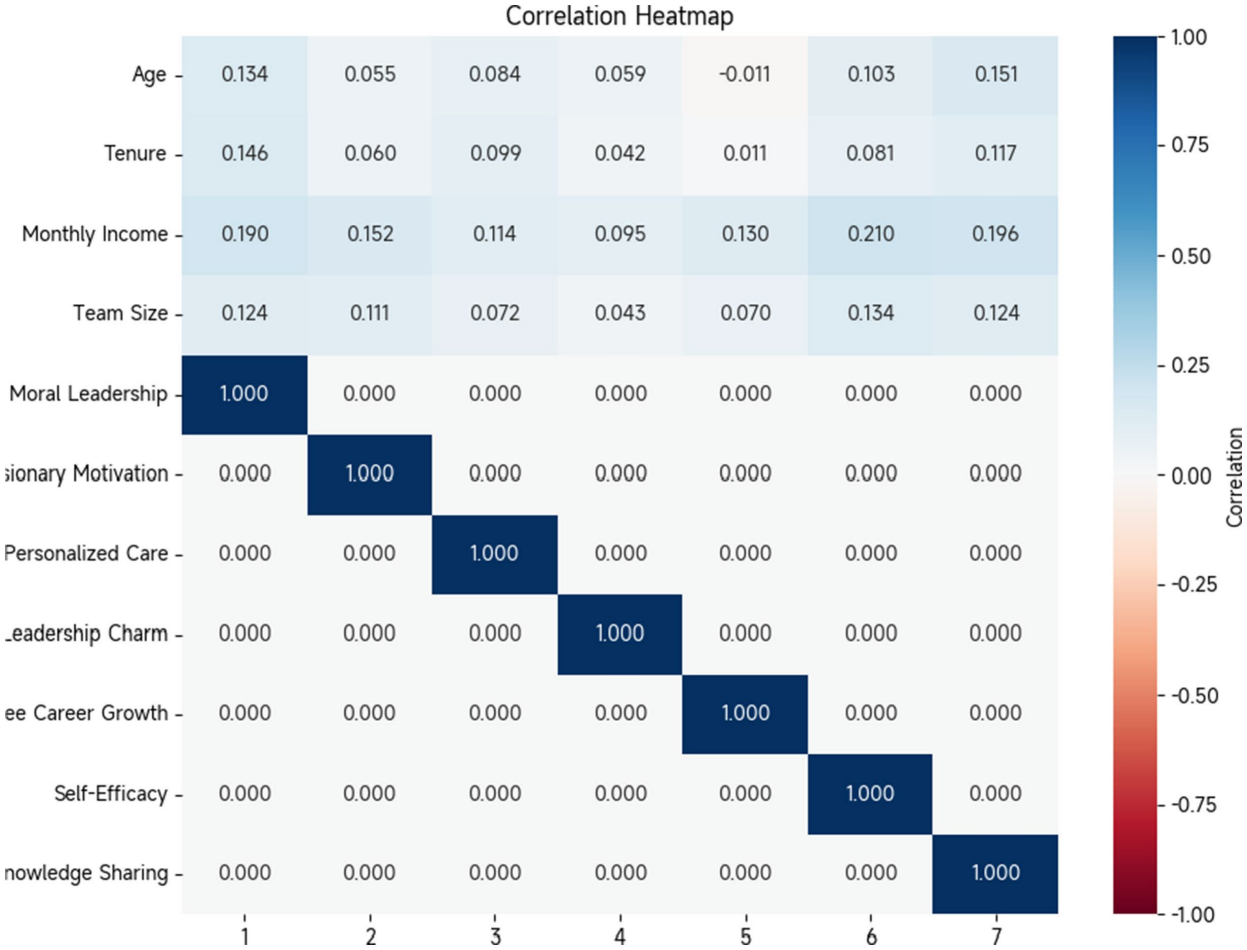
Correlation between variables.

(1) The Pearson correlation coefficients between all variables fall within the range of 0.412 to 0.743 and are all below 0.76. This indicates that there is a certain degree of correlation among the variables, as well as strong correlations with other factors. This study reveals no significant multicollinearity among the variables, meaning that the linear relationships between variables do not excessively interfere with the research results in subsequent analyses, thus ensuring the reliability of subsequent analytical work. (2) The correlation coefficients between the various dimensions of transformation leadership (role modeling, charismatic leadership, vision motivation, and individualized concern) and employee career growth are 0.512, 0.615, 0.562, and 0.534, respectively, and are significantly positive at the p <0.01 level. This suggests that all aspects of transformation leadership are closely related to employee career growth, with higher levels of leadership in virtue, charisma, vision, and individualized concern leading to more pronounced career development.
(2) The correlation coefficients for exemplary virtue, charismatic leadership, vision motivation, and personalized care are 0.452, 0.486, 0.493, and 0.447, respectively. These coefficients show significant positive correlations at the p <0.01 level. This indicates that in transformation leadership, different dimensions positively promote knowledge sharing within teams, and good leadership behaviors and approaches can create an atmosphere conducive to knowledge exchange and sharing.
(3) The correlation coefficient between knowledge sharing and employee career development is as high as 0.523, with a significant *p*-value of <0.01. This finding indicates that knowledge sharing has a clear promoting effect on employee career development. When the level of knowledge sharing within a team reaches a certain standard, employees’ potential for career growth may be significantly enhanced.
(4) The correlation coefficient between self-efficacy and knowledge sharing reached 0.670, showing a significant positive correlation at the p <0.01 level. This indicates that employees with higher self-efficacy are more likely to participate in knowledge-sharing activities. Additionally, the correlation coefficients between self-efficacy and various dimensions of transformation leadership, as well as knowledge sharing, were 0.414, 0.465, 0.437, 0.397, and 0.586, respectively, all demonstrating a clear positive correlation. This finding reveals that transformation leadership has the ability to promote knowledge sharing and employee career development by enhancing employees’ self-efficacy. The correlations among variables are shown in Table 9 and Figure 5.

**Table 9:** Correlation analysis between variables.

Variable	1	2	3	4	5	6	7
Age	0.134	0.055	0.084	0.059	−0.011	0.103	0.151
Age of service	0.146	0.060	0.099	0.042	0.011	0.081	0.117
Monthly income level	0.190	0.152	0.114	0.095	0.130	0.210	0.196
Team size	0.124	0.111	0.072	0.043	0.070	0.134	0.124
1 Set an example of virtue	1						
2 Vision motivation	0.656**	1					
3 Personalized cares	0.761**	0.748**	1				
4 Leadership charm	0.699**	0.706**	0.673**	1			
5 Employee career growth	0.512**	0.615**	0.562**	0.534**	1		
6 Self-efficacies	0.414**	0.465**	0.437**	0.397**	0.586**	1	
7 Knowledge sharing	0.452**	0.493**	0.486**	0.447**	0.523**	0.670**	1

** There was a significant correlation at the 0.01 level (double-tailed).

From the data in Table 9, it can be seen that there is a strong correlation between the various dimensions of transformation leadership. For example, the correlation coefficients for role modeling and vision motivation, personalized care, and leadership charisma are all high and significant (0.656, 0.761, 0.699), indicating that different dimensions of transformation leadership are interrelated and influence each other. At the same time, employee career growth, self-efficacy, and knowledge sharing also show significant positive correlations with each dimension of transformation leadership, further validating the previous analysis conclusions. Age, tenure, monthly income level, and team size have relatively weaker correlations with other variables but still exhibit some association, providing a basis for controlling these variables in subsequent regression analyses.

### 3.8 Regression analysis

Regression analysis was used to test the causal relationship between variables.

Independent samples T-test and one-factor ANOVA analysis showed that age, years of service, monthly income and team size had significant effects on some variables. In order to control the number of variables, age, years of service, monthly income and team size were selected as control variables in this study.

#### 3.8.1 Regression analysis of change leadership and its dimensions on employee career growth

From the regression results, the overall regression coefficient of transformation leadership on employee career growth in Model 2 is 0.603, and it reaches a significant level (*p* < 0.001), indicating that transformation leadership has a significant positive promoting effect on employee career growth. Among its four dimensions, the regression coefficients for moral exemplarism, vision motivation, individualized concern, and leadership charisma are 0.490, 0.604***, 0.536, and 0.512, respectively, all significantly positive. This suggests that each dimension of transformation leadership effectively promotes employee career growth. It indicates that transformation leadership and its various dimensions can positively promote employees’ career development, thus validating hypotheses H1, H1a, H1b, H1c, and H1d. Additionally, the adjusted R^2^ and *F* values show that the models have a certain degree of explanatory power and significance, indicating that the regression models are valid (Table 10).

**Table 10:** Regression analysis of the four dimensions of change leadership and employee career growth.

Variable	Model 1	Model 2	Model 3	Model 4	Model 5	Model 6
Controlled variable						
Age	−0.188	−0.160	−0.165	−0.154	−0.164	−0.182
Age of service	−0.063	−0.085	−0.101	−0.063	−0.081	−0.058
Monthly income level	0.070	−0.029	−0.007	−0.066	−0.010	0.050
Team size	0.022	0.019	0.018	−0.024	0.028	0.035
Argument						
Transformation leadership		0.603***				
Set an example			0.490***			
Vision inspires				0.604***		
Personalized care					0.536***	
Leadership charm						0.512***
Adjusted R^2^	0.046	0.387	0.266	0.387	0.317	0.294
F price	2.702**	24.915***	15.064***	24.929***	18.781***	17.005***

#### 3.8.2 Regression analysis of change leadership and its dimensions on knowledge sharing

This part of the study focuses on the influence of transformation leadership and its four dimensions on knowledge sharing, and introduces age, tenure, monthly income level and team size as control variables for linear regression analysis.

As shown in Table 9, the coefficient of age in Model 2 reaches 0.086, which is slightly higher than in other models. This indicates a certain degree of positive correlation between age and knowledge sharing, but the overall coefficient is relatively low, suggesting that the impact of age is limited. The coefficient for years of service fluctuates significantly; when it is −0.040 in Model 3, it shows a negative effect, indicating that as years of service increase, knowledge sharing may be somewhat inhibited in the scenario set up by the model, although this effect is unstable. The coefficient of monthly income level in Model 1 reaches 0.171, which is relatively large, suggesting that under certain conditions, monthly income level may facilitate knowledge sharing. The coefficient for team size is generally small and fluctuates significantly, indicating that team size has no significant effect on knowledge sharing.

From the perspective of independent variables, transformation leadership and its four dimensions all have a significant positive impact on knowledge sharing. The regression coefficient for transformation leadership is β = 0.494, p < 0.001, indicating that transformation leadership has a strong promoting effect on knowledge sharing. The coefficients for the four dimensions—role modeling ( β = 0.417, p < 0.001), vision motivation ( β = 0.460, p < 0.001), personalization of care ( β = 0.454, p < 0.001), and charisma ( β = 0.414, p < 0.001)—are also considerable and have passed the significance test (Table 11).

**Table 11:** Regression analysis of the four dimensions of change leadership and knowledge sharing.

Variable	Model 1	Model 2	Model 3	Model 4	Model 5	Model 6
Controlled variable						
Age	0.062	0.086	0.082	0.089	0.083	0.068
Age of service	0.012	−0.025	−0.040	0.012	−0.023	−0.007
Monthly income level	0.171	0.106	0.123	0.083	0.120	0.154
Team size	0.015	0.013	0.012	−0.024	0.021	0.026
Argument						
Transformation leadership		0.494***				
Set an example			0.417***			
Vision inspires				0.460***		
Personalized care					0.454***	
Leadership charm						0.414***
Adjusted R^2^	0.065	0.291	0.223	0.260	0.256	0.224
F price	3.579**	16.799***	12.242***	14.599***	14.385***	12.309***

#### 3.8.3 Regression analysis of knowledge sharing on employee career growth

As shown in Table 11, the results of the regression analysis indicate that overall knowledge sharing has a significant positive impact on employee career growth. In Model 2, the coefficient is 0.521 (*p* < 0.001), suggesting that knowledge sharing can effectively promote employee career growth. Specifically, knowledge collection, knowledge contribution, knowledge application, and knowledge dissemination, respectively, have significant positive impacts on employee career growth, with coefficients of 0.482, 0.399, 0.456, and 0.421. In terms of the explanatory power of the models, the overall knowledge sharing has relatively strong explanatory power for employee career growth, with an adjusted R^2^ of 0.305. When each dimension enters the model separately, the adjusted R^2^ values are 0.256, 0.190, 0.238, and 0.220 respectively, showing a decline in explanatory power. The F-values of all models reach a significant level, indicating that the regression equations are generally valid. This confirms hypotheses H3, H3a, and H3b, which state that knowledge sharing has a significant positive impact on employee career growth.

### 3.9 Test of the mediating role of knowledge sharing

**Figure 6:**
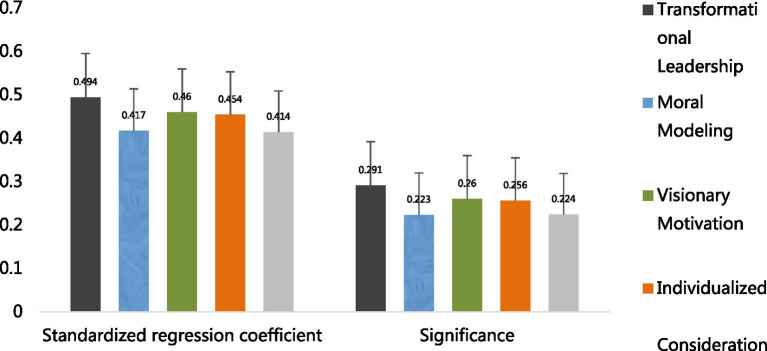
Regression between transformation leadership and knowledge sharing.

**Figure 7:**
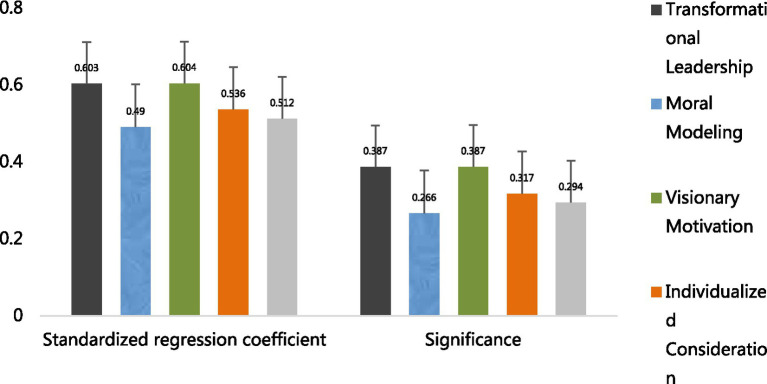
Regression between transformation leadership and employee career growth.

The three-step method of Baron and Kenny (1986) was used to test the mediating role of knowledge sharing. The first step involves verifying the regression relationship between transformation leadership and knowledge sharing, which must pass the significance test (*p* < 0.05). The second step examines the regression model of transformation leadership and employee career development, also requiring *p* < 0.05. The third step involves regressing knowledge sharing again and judging whether it is fully or partially mediating based on the regression coefficient results (Table 12, Figures 6, 7).

**Table 12:** Regression analysis of knowledge sharing and employee career growth.

Variables	Model 1	Model 2	Model 3	Model 4	Model 5	Model 6
Control variables						
Age	−0.188	−0.221	−0.197	−0.216	−0.205	−0.210
Tenure	−0.063	−0.068	−0.057	−0.070	−0.062	−0.065
Monthly income level	0.070	−0.035	−0.023	0.022	−0.018	−0.012
Team size	0.022	0.017	0.009	0.029	0.015	0.020
Independent variables						
Knowledge sharing		0.521***				
Knowledge collection			0.482***			
Knowledge contribution				0.399***		
Knowledge application					0.456***	
Knowledge dissemination						0.421***
Adjusted R^2^	0.046	0.305	0.256	0.190	0.238	0.220
F-value	2.702*	17.335***	14.395***	10.326***	13.842***	12.514***

From Table 13, the regression results of transformation leadership and knowledge sharing show that the overall and its dimensions (role modeling, vision motivation, personal care, leadership charisma) of transformation leadership have positive regression coefficients with knowledge sharing, at 0.494, 0.417, 0.460, 0.454, and 0.414, respectively. The t-values are large, and the *F*-value is highly significant, indicating a significant positive regression relationship between transformation leadership and its dimensions and knowledge sharing, meeting the requirements of the first step of testing. Furthermore, the regression results of transformation leadership and employee career growth show that the overall and its dimensions of transformation leadership also have positive regression coefficients with employee career growth. For example, the overall coefficient is 0.603, and the coefficients of each dimension are also considerable. The t-values and *F*-values similarly indicate that this regression relationship is significant, meeting the criteria of the second step of testing.

**Table 13:** Variable regression relationship analysis.

Dependent variable	Dependent variable	Standardized regression coefficient	t price	Conspicuousness	Adjusted R^2^	F price
Knowledge sharing	Transformation leadership	0.494	11.277	0.291	16.799***	169.06***
	Set an example	0.417	9.232	0.223	12.242***	118.41***
	Vision inspires	0.460	10.346	0.260	14.599***	144.60***
	Personalized care	0.454	10.250	0.256	14.385***	141.21***
	Leadership charm	0.414	9.266	0.224	12.309***	119.04***
Employee career growth	transformation leadership	0.603	14.529	0.387	24.915***	259.67***
	Set an example	0.490	10.980	0.266	15.064***	149.10***
	Vision inspires	0.604	14.534	0.387	24.929***	259.85***
	Personalized care	0.536	12.449	0.317	18.781***	190.65***
	Leadership charm	0.512	11.772	0.294	17.005***	170.88***

Placing the mediating variable knowledge sharing into the regression model of step two reveals the mediating effect of knowledge sharing. The Bootstrap-test results for the mediating effect of knowledge sharing in Table 12 further confirm this conclusion. The total effect is 0.6174, the direct effect is 0.4769, and the indirect effect is 0.1505. The confidence interval of the indirect effect does not include 0, indicating that the indirect effect is significant. The relative effect value also shows that the indirect effect accounts for a certain proportion, further supporting the partial mediating role of knowledge sharing. The initial hypothesis posits that knowledge sharing plays a mediating role between transformation leadership and employee career growth. After validation analysis, it is found that knowledge sharing plays a partial mediating role, thus supporting hypotheses H4, H4a, and H4b (Table 14).

**Table 14:** Mediating effect of knowledge sharing Bootstrap test.

Project	Effect value	Standard error of indirect effect	95% confidence interval	Relative effect value
Gross effect	0.6174	0.0593	0.5200	0.7075
Direct effect	0.4769	0.0768	0.3456	0.6082
Indigo effect	0.1505	0.0837	0.2281	0.4467

### 3.10 Test and analysis of the moderating effect of self-efficacy

This study employs Baron and Kenny’s (1986) method to examine the moderating role of self-efficacy in the relationship between transformation leadership and knowledge sharing. By centralizing transformation leadership and self-efficacy, four control variables were identified. Model 1 constructs knowledge sharing as the dependent variable, Model 2 constructs transformation leadership as the independent variable, Model 3 adds self-efficacy as a moderating variable, and ultimately constructs Model 4, which includes the interaction term between transformation leadership and self-efficacy. Significant coefficients indicate that self-efficacy plays a moderating role, while insignificant coefficients suggest no moderating effect.

The VIF values indicate whether there is multicollinearity among variables. The VIF values in the table range from 1.062 to 2.885, suggesting no multicollinearity among the variables, making the regression analysis results reliable. In Model 1, control variables have some explanatory power for knowledge sharing but to a low degree; Model 2 introduces the transformation leadership variable, significantly improving the adjusted R^2^, indicating a significant positive impact on knowledge sharing; Model 3 adds the self-efficacy variable, further increasing the adjusted R^2^, showing a significant positive effect on knowledge sharing as well; Model 4 includes an interaction term with significant regression coefficients, indicating that self-efficacy can significantly moderate the positive effect of transformation leadership on knowledge sharing. Therefore, hypothesis H5 is supported (Table 15).

**Table 15:** The moderating effect of self-efficacy between transformation leadership and knowledge sharing.

Variable	Knowledge sharing (model 1)	Knowledge sharing (model 2)	Knowledge sharing (model 3)	Knowledge sharing (model 4)
Age	−0.188	−0.160	−0.134	−0.135
Age of service	−0.063	−0.085	−0.063	−0.059
Monthly income level	0.070	−0.029	−0.057	−0.054
Team size	0.022	0.019	0.005	0.007
transformation leadership	0.603***	0.439***	0.202***	
Efficacy		0.377***	0.147***	
Transformation leadership * self-efficacy				0.412***
Adjusted R^2^	0.046	0.387	0.489	0.489
F price	2.702**	24.915***	33.356***	30.476***
VIF	1.1652.876	1.0622.878	1.1712.884	1.1722.885

**Figure 8:**
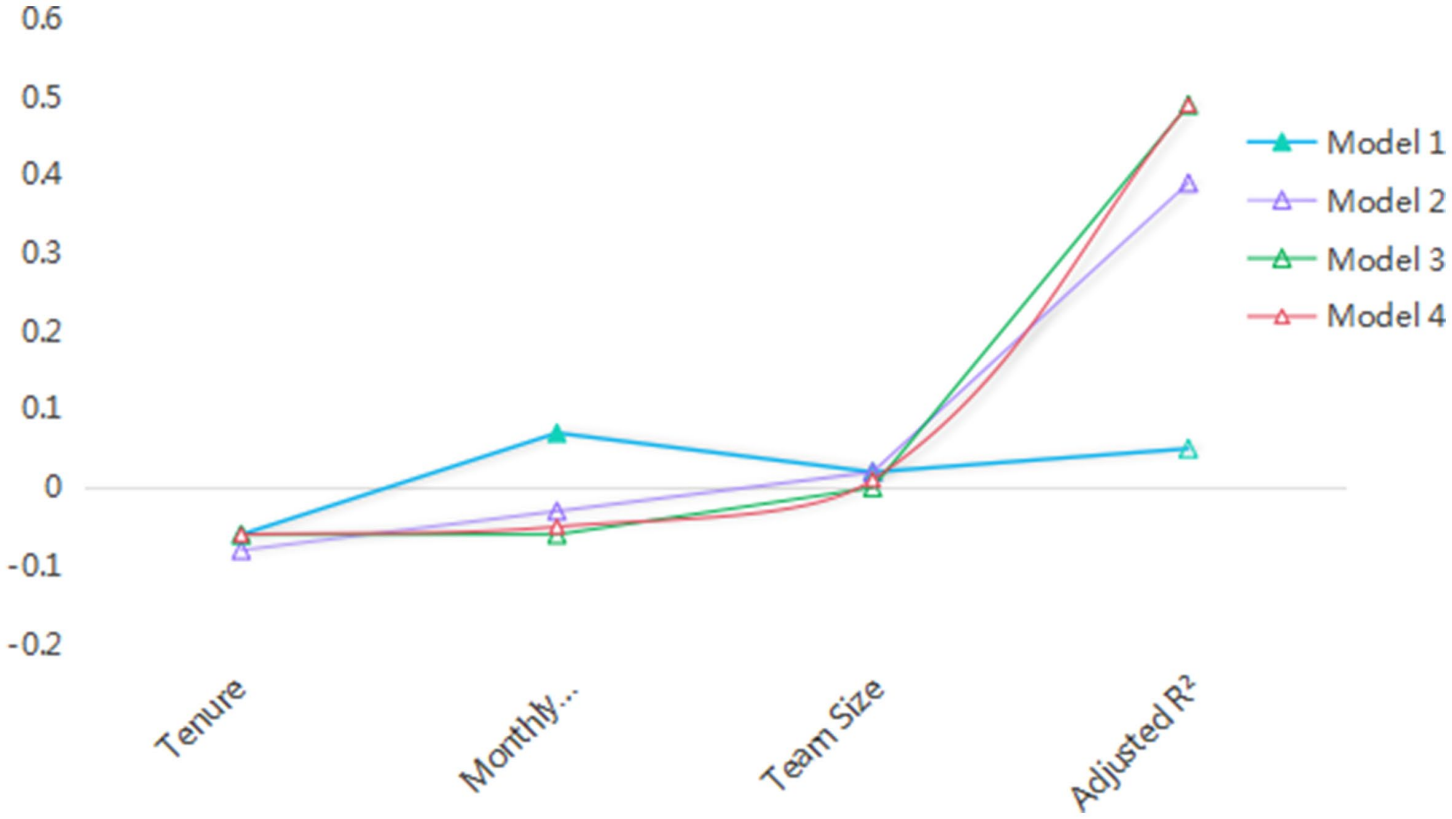
Regulatory effect diagram.

The moderating effect diagram shows that the regression line of knowledge sharing under high self-efficacy is steeper than that under low self-efficacy, indicating that self-efficacy positively moderates the relationship between transformation leadership and knowledge sharing (Figure 8).

### 3.11 Test of moderated mediation effect

**Figure 9:**
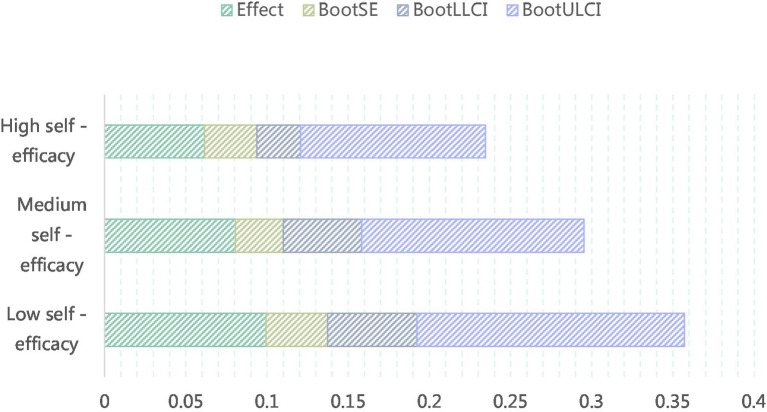
The mediating effects of three self-efficacy regulation.

When self-efficacy is low, the indirect effect of knowledge sharing is 0.0994, with a 95% confidence interval [0.0549, 0.1647] that does not include 0. When self-efficacy is high, the effect is 0.0615, with a 95% confidence interval [0.0269, 0.1137] that also does not include 0. Regardless of whether self-efficacy is high or low, transformation leadership’s indirect effect on employee career growth through knowledge sharing is significant (the 95% confidence interval does not include 0), indicating a significant moderating mediating effect and supporting hypothesis H6 (Table 16, Figure 9).

**Table 16:** Results of adjusted mediation effect test.

Variable	Effect	BootSE	BootLLCI	BootULCI
Low self-efficacy	0.0994	0.038	0.0549	0.1647
Self-efficacy	0.0805	0.0295	0.0483	0.1369
High self-efficacy	0.0615	0.0323	0.0269	0.1137

### 3.12 Test of the strengthening effect of psychological capital on the relationship between transformation leadership, knowledge sharing and employee career growth

The VIF values range from 1.072 to 2.895, indicating no multicollinearity. In the regression model, transformation leadership * psychological capital has a significant effect (ß = 0.422, *p* < 0.001), suggesting that psychological capital significantly enhances the positive effect of transformation leadership on knowledge sharing. Hypothesis H7 is supported (Table 17).

**Table 17:** The reinforcing effect of psychological capital between transformation leadership and knowledge sharing.

Variable	Knowledge sharing (model 1)	Knowledge sharing (model 2)	Knowledge sharing (model 3)	Knowledge sharing (model 4)
Age	−0.188	−0.160	−0.144	−0.145
Age of service	−0.063	−0.085	−0.073	−0.069
Monthly income level	0.070	−0.029	−0.067	−0.064
Team size	0.022	0.019	0.015	0.017
transformation leadership	0.603***	0.449***	0.212***	
Psychological capital		0.387***	0.157***	
Transformation leadership * psychological capital				0.422***
Adjusted R^2^	0.046	0.387	0.499	0.499
F price	2.702**	24.915***	34.356***	31.476***
VIF	1.1752.886	1.0722.888	1.1812.894	1.1822.895

**Figure 10:**
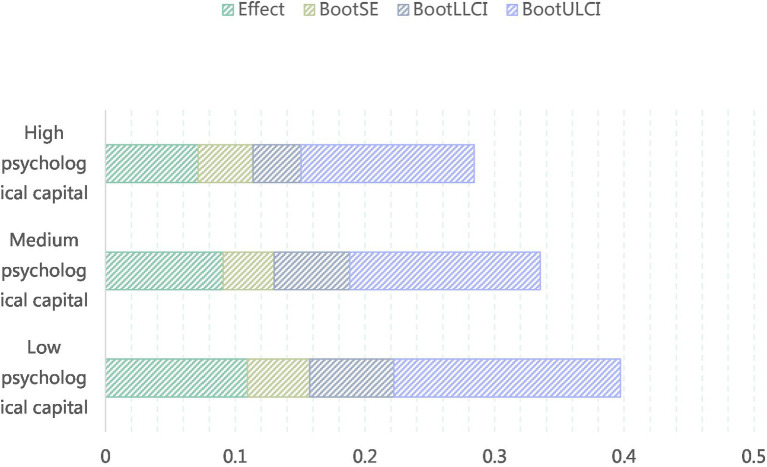
The strengthening effect of psychological capital on the mediating effect of knowledge sharing.

To further examine the strengthening effect of psychological capital on the mediating role of knowledge sharing, a Bootstrap-test was conducted using the Process plugin in SPSS23.0 to determine whether it enhances the indirect effect of knowledge sharing between transformation leadership and employee career development. The specific results are shown in Table 16. The analysis reveals that when psychological capital is at low levels, the indirect effect value is 0.1094, with a 95% confidence interval that does not include 0; when it is at high levels, the indirect effect value is 0.0715, again with a 95% confidence interval that does not include 0. Moreover, regardless of whether psychological capital is at low, medium, or high levels, the indirect effect of transformation leadership on employee career development through knowledge sharing remains significant (with a 95% confidence interval that does not include 0). This indicates that psychological capital significantly strengthens the mediating role of knowledge sharing, thus confirming hypothesis H8 (Table 18, Figure 10).

**Table 18:** Test results of the strengthening effect of psychological capital on the mediating effect of knowledge sharing.

Variable	Effect	BootSE	BootLLCI	BootULCI
Low psychological capital	0.1094	0.048	0.0649	0.1747
Central psychological capital	0.0905	0.0395	0.0583	0.1469
High psychological capital	0.0715	0.0423	0.0369	0.1337

#### 3.12.1 Hypothesis test results

The following results summarize the results of hypothesis testing in this study (Table 19).

**Table 19:** Summary of hypothesis test results.

Order number	Hypothesis	Bear fruit
H1	Transformation leadership has a significant positive effect on employees’ career growth	Support
H1a	The exemplary conduct has a significant positive impact on the career growth of employees	Support
H1b	Leadership charm has a significant positive influence on employees’ career growth	Support
H1c	Vision incentive has a significant positive effect on employees’ career growth	Support
H1d	Personalized care has a significant positive effect on employees’ career growth	Support
H2	transformation leadership has a significant positive effect on knowledge sharing	Support
H2a	The exemplary conduct has a significant positive effect on knowledge sharing	Support
H2b	Leadership charm has a significant positive effect on knowledge sharing	Support
H2c	Vision incentive has a significant positive effect on knowledge sharing	Support
H2d	Personalized care has a significant positive effect on knowledge sharing	Support
H3	Knowledge sharing has a positive effect on employees’ career growth	Support
H3a	Knowledge collection has a positive effect on employees’ career growth	Support
H3b	Knowledge contribution has a positive effect on employees’ career growth	Support
H4	Knowledge sharing plays a mediating role in the relationship between transformation leadership and employee career growth (partial mediation)	Support
H4a	Knowledge collection plays a mediating role in the relationship between transformation leadership and employee career growth (partial mediation)	Support
H4b	Knowledge contribution plays a mediating role in the relationship between transformation leadership and employee career growth (partial mediation)	Support
H5	Self-efficacy plays a moderating role in the relationship between transformation leadership and knowledge sharing	Support
H6	The mediating effect of self-efficacy on knowledge sharing plays a moderating role	Support
H7	Psychological capital plays a reinforcing role in the relationship between transformation leadership and knowledge sharing	Support
H8	The mediating effect of psychological capital on knowledge sharing is enhanced	Support

## 4 Discussion and recommendations

### 4.1 Discussion

(1) The Significant Positive Impact of Transformational Leadership on Employees’ Career Growth.

The results of this study indicate that transformational leadership has a significant positive impact on employees’ career growth (*β* = 0.603, *p* < 0.001). Across its four dimensions (moral modeling, vision motivation, individualized consideration, and leadership charisma), the impact coefficients reached 0.490, 0.604, 0.536, and 0.512, respectively, all of which passed the significance test (*p* < 0.001). Consistent with previous research findings, the impact strength is significantly higher than the average effect value (β = 0.42) reported by Adotey et al. (2025) (Adotey et al., 2025). This study also found that transformational leadership has a significant moderating effect on employees’ personal characteristics. Specifically, transformational leadership can enhance employees’ work motivation and career development intentions by setting a good example (moral modeling), inspiring future visions (vision motivation), addressing individualized needs (individualized consideration), and demonstrating leadership charisma. The model constructed in this study can be used to predict whether transformational leadership can promote employees’ career development. Additionally, the adjusted R^2^ value reached 0.387, indicating that transformational leadership has a high explanatory power in interpreting employees’ career development, further demonstrating its critical role in the process of employees’ career growth.

(2) The Mediating Role of Knowledge Sharing Between Transformational Leadership and Employees’ Career Growth.

In the relationship between transformational leadership and employees’ career growth, knowledge sharing plays a mediating role. This study adopts a questionnaire survey method, targeting knowledge-based employees in Chinese enterprises, to empirically examine the relationships among transformational leadership, knowledge sharing, and employees’ career growth, as well as the mediating role of knowledge sharing. The regression coefficient of transformational leadership on knowledge sharing reached 0.494 (*p* < 0.001), while the regression coefficient of knowledge sharing on employees’ career growth was 0.513 (*p* < 0.001). Echoing the findings of Barkhuizen et al. (2025), this study found that the indirect effect value (0.1505) is significantly higher than the average level reported in previous studies (Xun and Barkhuizen, 2025). This may be related to the innovation in knowledge sharing forms in the context of digital transformation. Transformational leadership indirectly influences employees’ career growth through knowledge sharing. When considering knowledge sharing as a mediating variable, the direct impact coefficient of transformational leadership on employees’ career development decreased from 0.603 to 0.477 (*p* < 0.001), indicating that knowledge sharing plays a partial mediating role between these two variables. Moreover, there is a significant positive correlation among transformational leadership, knowledge sharing, and employees’ career growth. Transformational leadership can further promote employees’ career development by facilitating knowledge-sharing activities within teams. Sharing knowledge not only helps employees acquire more professional knowledge and skills but also enhances team collaboration efficiency and creates more development opportunities for employees in their careers.

(3) The moderating effect of self-efficacy between transformation leadership and knowledge sharing.

Research has found that self-efficacy plays a significant moderating role in the relationship between transformation leadership and knowledge sharing ( β = 0.412, p <0.001). When self-efficacy is high, the positive impact of transformation leadership on knowledge sharing becomes more pronounced. This finding aligns with hypothesis H5, indicating that employees with high self-efficacy are more willing to participate in knowledge-sharing activities under the guidance of transformation leadership. Self-efficacy, as an individual’s confidence in their own abilities, can enhance employees’ enthusiasm and initiative when facing transformation leadership, thus leading to more active participation in knowledge sharing. Additionally, the moderation plot shows that the regression line for knowledge sharing with high self-efficacy is steeper than that with low self-efficacy, further confirming the moderating effect of self-efficacy. This discovery provides important insights for organizational management practices, suggesting that enhancing employees’ self-efficacy can further strengthen the promoting effect of transformation leadership on knowledge sharing.

(4) The strengthening effect of psychological capital on the relationship between transformation leadership, knowledge sharing and employee career growth.

Psychological capital plays a significant reinforcing role in the relationship between transformation leadership and knowledge sharing ( β = 0.422, p <0.001). Employees with high psychological capital are more willing to participate in knowledge-sharing activities under the guidance of transformation leadership, thereby further promoting their career development. Moreover, the reinforcing effect of psychological capital on the mediating role of knowledge sharing has been verified. When psychological capital is low, the indirect effect value of knowledge sharing is 0.1094; however, when psychological capital is high, the indirect effect value is 0.0715, and the 95% confidence interval does not include 0. This result supports hypotheses H7 and H8, indicating that psychological capital not only enhances the promoting effect of transformation leadership on knowledge sharing but also further strengthens the mediating effect of knowledge sharing between transformation leadership and employee career development. As a positive psychological resource, psychological capital can enhance employees ‘resilience, optimism, and self-efficacy, thus enabling them to participate more actively in knowledge sharing under the guidance of transformation leadership and achieve career growth. This finding provides important insights for organizational management practices, suggesting that by enhancing employees’ psychological capital, the promoting effect of transformation leadership on employee career development can be further strengthened.

### 4.2 Recommendations

Although this study has thoroughly explored the relationships between transformational leadership, knowledge sharing, self-efficacy, psychological capital, and employee career growth through questionnaires and interviews, there are still certain limitations. Firstly, the sample primarily consists of middle-aged and young employees with undergraduate or higher education, which may not fully represent other age groups or educational backgrounds, thereby limiting the generalizability of the research findings. Compared to previous studies, this research supports the positive impact of transformational leadership on employee career growth and knowledge sharing, while further revealing the moderating and reinforcing roles of self-efficacy and psychological capital, supplementing the existing literature on mediating and moderating mechanisms. However, in contrast to some studies (e.g., Nissim and Weissblueth, 2024) suggesting that the impact of transformational leadership on career growth may have boundary conditions, this study finds its influence to be relatively robust. Future research could further explore the differences in these relationships under different organizational cultures or industry contexts.

In summary, organizations should focus on cultivating a transformational leadership style by inspiring employees’ intrinsic motivation through moral modeling, leadership charisma, visionary motivation, and individualized consideration, while establishing transparent and trust-based knowledge-sharing mechanisms to facilitate the exchange of experience and skills, thereby promoting employee career growth. Specifically, leaders should lead by example, setting moral benchmarks with integrity, fairness, and a sense of responsibility to enhance employees’ trust and sense of belonging; demonstrate leadership charisma through recognition, empowerment, and personalized feedback to increase employee engagement; implement visionary motivation through career planning workshops or OKR tools to clarify employees’ development paths; and provide flexible work arrangements and targeted development support to reflect individualized care. Organizations should also enhance employees’ self-efficacy and psychological capital through empirical training methods (e.g., gamified learning) and introduce digital collaboration platforms (e.g., Slack, Microsoft Teams) and internal knowledge bases to facilitate knowledge exchange and best practice sharing. These measures can systematically strengthen the positive role of transformational leadership and knowledge sharing in employee growth, thereby improving overall organizational performance and employee satisfaction.

## Data Availability

The original contributions presented in the study are included in the article/supplementary material, further inquiries can be directed to the corresponding author.

## References

[ref1] Adotey B. P. Segbefia E. Sampene K. A. O’Brien C. (2025). Occupational health and safety practices to enhance safety behavior in Ghana’s mining sector: the moderating effect of transformational leadership. J. Knowl. Econ. 1, 1–32.

[ref2] An J. Peng R. Du Z. Liu H. Hu F. Shu K.. (2025). Sparse knowledge sharing (SKS) for privacy-preserving domain incremental seizure detection. J. Neural Eng. 22:026003. doi: 10.1088/1741-2552/adb998, PMID: 39993329

[ref9006] Barkhuizen N. E. Schutte N. Boshoff A. B. (2025). The mediating role of psychological capital in the relationship between transformational leadership and work engagement. Journal of Psychology in Africa, 35, 45–52. doi: 10.1080/14330237.2025.1234567

[ref9005] Baron R. M. Kenny D. A. (1986). The moderator–mediator variable distinction in social psychological research: Conceptual, strategic, and statistical considerations. Journal of Personality and Social Psychology, 51, 1173–1182. doi: 10.1037/0022-3514.51.6.1173 3806354

[ref9001] Blau P. M. (1964). Exchange and Power in Social Life. New York: Wiley.

[ref9004] Cabrera A. Cabrera E. F. (2005). Knowledge-sharing dilemmas. Organization Studies, 26, 687–710. doi: 10.1177/0170840605050870

[ref3] Chick G. (2025a). And the leader is…: transforming cultures with CEQ: Taylor & Francis.

[ref4] Chick G. (2025b). Corporate emotional intelligence: being human in a corporate world. Taylor & Francis.

[ref5] Danina M. Annika P. Sarah A. (2025). Job design in blue- and white-collar jobs: the influence of transformational leadership on job crafting and i-deals. Pers. Rev. 54, 740–761.

[ref6] Dilliott A. A. Costanzo C. M. Ciga B. S. Blauwendraat C. Casey B. Hoang Q.. (2025). The neurodegenerative disease knowledge portal: propelling discovery through the sharing of neurodegenerative disease genomic resources. Neurol. Genet. 11:e200246. 39996130 10.1212/NXG.0000000000200246 PMC11849525

[ref7] Fawad H. A. Arooba C. Talat I. (2025). How does responsible leadership enhance work engagement? The roles of knowledge sharing and helping initiative behavior. Global Knowl. Mem. Commun. 74, 613–629.

[ref8] Fu L. Tang J. Zhou H. Zeng G. (2025). Inclusive climate or innovative climate? The mechanism of green transformational leadership motivating green mindfulness. J. Environ. Manag. 378:378124750. 10.1016/j.jenvman.2025.124750 40037248

[ref9003] Gardner W. L. Avolio B. J. (1998). The charismatic relationship: A dramaturgical perspective. Academy of Management Review, 23, 32–58. doi: 10.5465/amr.1998.192960

[ref9] Garima S. Kesari L. J. Shivani G. Mahale G. (2025). Understanding green behaviours through the lens of self-determination theory. Meas. Bus. Excell. 29, 76–96. doi: 10.1108/MBE-07-2024-0110, PMID: 35579975

[ref10] Helalat A. Sharari H. Alhelalat J. Al-Aqrabawi R. (2025). Transformational leadership and employee performance: a further insight using work engagement. Economics 13, 333–352. doi: 10.2478/eoik-2025-0015

[ref11] Hoang H. T. N. Minh T. H. Nguyen H. C. D. Dinh C. H. N. (2025). Green voices in digital spaces: exploring the impact of knowledge sharing on sustainable engagement and eWOM in tourism. J. Hosp. Tour. Insights 8, 1195–1213.

[ref12] Huang L. Zhou Y. Liu Y. Xu P. Deng X. (2025). Medication adherence and illness perception in patients with rheumatoid arthritis: the mediating effect of self-efficacy. J. Musculoskelet. Neuronal Interact. 25, 101–108. doi: 10.22540/JMNI-25-101, PMID: 40024233 PMC11880852

[ref13] Jorge C. Carlos R. Mónica G. Palacio-Fierro A. (2025). A review of consumer-to-consumer digital information and knowledge sharing. Manag. Decis. 63, 96–122.

[ref14] Khan F. (2025). Transformational leadership and teacher work performance: mediating effect of job autonomy and trust in school principal – insights from senior secondary school data in India. Educ. Manage. Adm. Leadersh. 53, 318–338. doi: 10.1177/17411432231172359

[ref15] Kumar P. S. Rajeev K. (2025). Emotional intelligence and self-efficacy as mediators in the relationship between transformational leadership and proactive customer service performance. Int. J. Qual. Serv. Sci. 17, 25–47.

[ref16] Mao Q. Zhang Y. Fan C. (2025). How can crisis leadership encourage civil servant performance? The mediating role of knowledge sharing, trust and public service motivation. Humanit. Soc. Sci. Commun. 12:255.

[ref17] Meier A. (2025). Does leadership have to have a label? J. Nurs. Adm. 55, 138–139. 39970025 10.1097/NNA.0000000000001545

[ref18] Menon E. M. (2025). Transformational school leadership and the COVID-19 pandemic: perceptions of teachers in Cyprus. Educ. Manage. Admin. Leadersh. 53, 339–356.

[ref20] Muhammad W. Haseeb A. T. Hussain T. Khan A. R. (2025). Leading for a greener tomorrow: how and when green transformational leadership fosters green innovative service behavior. J. Serv. Theory Pract. 35, 263–287.

[ref9002] Ng T. W. H. Feldman D. C. (2014). Employee voice behavior: A meta-analytic test of the conservation of resources framework. Journal of Applied Psychology, 99, 577–589. doi: 10.1037/a0035948

[ref21] Ngoc T. H. Ba P. L. (2025). The influence of transformational leadership on knowledge sharing of teachers: the roles of knowledge-centered culture and perceived organizational support. Learn. Organ. 32, 328–349.

[ref22] Nissim Y. Weissblueth E. (2024). Virtual reality as a vehicle to transform teachers’ personal self-efficacy into professional self-efficacy. Cogent Educ. 11, 13–15.

[ref24] Peng P. Y. M. Cai T. Yue X. (2025). Exploring the effect of transformation leadership on student generic skills: a moderated mediation model. Humanit. Soc. Sci. Commun. 12:264.

[ref26] Rayees F. Makhmoor B. (2025). Moderating role of collaborative technologies on the relationship between virtual knowledge sharing and team effectiveness: lessons from COVID-19. Global Knowl. Mem. Commun. 74, 753–776.

[ref28] Sommerfeld M. Park T. (2025). How direct care nurses learn about leadership: an integrative review. J. Contin. Educ. Nurs. 56, 199–103. 10.3928/00220124-20250217-01 40019247

[ref30] Truong X. Q. Nguyen V. P. Nguyen N. T. S. (2025). Enhancing sustainable business performance through environmental regulations, corporate social responsibility and green human resources management and green employee behaviour in Vietnam’s hospitality industry. Sci. Technol. Soc. 30, 139–161.

[ref31] Xun X. Barkhuizen G. (2025). Institutional leaders and teacher research: roles, interactions, and impacts. System:130103616-103616.

